# Reunion with a peer partner reduces PVN oxytocin neuron immunoreactivity in socially selective voles

**DOI:** 10.1038/s41598-025-17920-3

**Published:** 2025-09-29

**Authors:** Nastacia L. Goodwin, Al M. Licata, Kelley C. Power, Kara M. Reitz, Yasmin Kamal, Annaliese K. Beery

**Affiliations:** 1 Psychology Department, University of Washington, Seattle, WA 98105 USA; 2 Neuroscience Program, Smith College, Northampton, MA 01063 USA; 3 Neuroscience Program, Mount Holyoke College, Holyoke, MA USA; 4 Department of Integrative Biology, UC Berkeley, Berkeley, CA 94720 USA; 5 Department of Medicine, Division of Gastroenterology, University of Pennsylvania, Philadelphia, PA 19104 USA; 6 Department of Neuroscience, UC Berkeley, 3040 Valley Life Sciences Building # 3140, Berkeley, CA 94720-3140 USA

**Keywords:** Reunion, Social behavior, Oxytocin, cFos, Corticosterone, Meadow vole, Social behaviour, Social neuroscience

## Abstract

Friendships—i.e. selective peer relationships—are an important aspect of human behavior, but are rare in rodent species. Meadow voles are seasonally social rodents that form non-reproductive social groups in winter/short day lengths that are selective in nature. Across rodents, oxytocin neurons in the paraventricular nucleus (PVN) of the hypothalamus are typically active during socially salient events, including interaction with novel individuals as well as social separation. To assess whether familiar and novel peer interactions produce different patterns of immunolabeling in a species that forms bonds with familiar individuals, we measured oxytocin neuron immunoreactivity and colabeling with the immediate early gene product cFos. Oxytocin labeling and oxytocin/cFos colabeling were higher after interaction with a novel same-sex conspecific than after reunion with a peer partner. Colabeling was also high after 24 h separation without reunion. Circulating corticosterone concentrations paralleled PVN oxytocin neuron activity. We also investigated whether oxytocin signaling was photoperiod dependent and could contribute to seasonal differences in meadow vole social behavior. Oxytocin receptor densities are known to be higher in multiple brain regions in short day lengths in meadow voles, but we found no concomitant change in PVN oxytocin positive cell count. Together these studies indicate that seasonal changes in behavior correlate with oxytocin signaling at the receptor level, while short term experiences modulated oxytocin neuron activity differentially by social context.

**Supplementary Information:**

The online version contains supplementary material available at 10.1038/s41598-025-17920-3.

## Introduction

In humans and other social mammals, affiliative interactions with peers, romantic partners, and family members promote physical and mental health, as well as longevity^1–4^. In contrast, lack of social connection and perceived isolation are associated with negative health impacts^5,6^. While isolation is broadly detrimental for social mammals, renewed social contact ameliorates some of these consequences. However, what happens upon reunion with specific bonded companions has received little attention, in part because traditional laboratory rodent species don’t form stable relationships and prefer social novelty^7–11^. The nature of positive peer social interactions varies substantially by species, from gregarious interactions to selective social relationships with specific friends or peers. Multiple vole species form selective peer relationships^10,12^, providing opportunities to study peer relationship dynamics and familiarity preference.

Oxytocin (OT), a 9-amino acid neuropeptide and hormone, is well established as a mediator of diverse social behaviors from social reward to recognition^13–15^. Oxytocin is produced in the supraoptic nucleus (SON) and paraventricular nucleus (PVN) of the hypothalamus and released into the brain and peripheral circulation^16^. Oxytocin facilitates social bond formation in both parental relationships and mate partnerships^17–20^, and growing evidence supports OT’s role in mediating the selectivity of social relationships between peers. In humans and chimpanzees, OT reinforces in-group/out-group dynamics^21,22^, and OT shapes the selectivity of peer partner preferences and social reward in meadow voles and prairie voles^23–25^. In California mice, a population of OT neurons in the BNST mediates social avoidance^26^.

The impacts of peer social environments on oxytocin signaling have been most explored in the context of social stress (e.g. crowding, social defeat, and isolation), and social buffering of stress responses^27–30^. While not considered a traditional stress hormone, OT neurons are activated and OT is released in response to stress or cortisol/corticosterone exposure^31–35^ and OT plays an important role in reducing stress responses^36–38^.

In humans, acute isolation leads to increased midbrain responses to social cues, similar to hunger^39^. In mice, PVN OT appears to mediate social craving^40,41^. Isolation activates OT neurons^41^, OT/cFos colabeling in the PVN is increased after 1 week of separation^42^, and chemogenetic activation of PVN OT + neurons during isolation promotes social interaction during reunion^41^.

In prairie voles, separation from a mate or sibling leads to depression-like behavioral symptoms^43–46^, physiological indicators of reduced health^47–49^, and changes in oxytocin signaling. Extended separation from a sibling leads to increased circulating OT, and increased OT/cFos positive cell counts in the PVN of females^44,50^. Similarly, increased OT mRNA was present in the PVN of young males following post-weaning isolation^51^. Oxytocin administration also protects against the negative health consequences of isolation^49,52^.

While isolation increased PVN oxytocin signaling in the above studies, other interactions with high social salience increase PVN OT neuron activity and neural OT release. Microdialysis studies indicate that OT is released into specific brain regions during reproductive activities such as mating, parturition and suckling^53–55^, as well as during aggressive interactions^56^. Aggressive interactions also increase OT/cFos colabeling in the PVN in both mice and prairie voles^44,57^. Other studies indicate that PVN OT neuron firing rates increase in males during presentation of an anesthetized stimulus mouse^58^, in virgin females during social learning of alloparental behaviors^59^, and OT neuron activation is also associated with play duration in young rats^60^. This diversity of releasing stimuli and associated outcomes underscores that activation of oxytocin receptors in different circuits may have different behavioral effects. Because PVN OT neurons are activated by multiple socially salient events, we assessed whether there are differences in OT neuron activity under conditions of familiar peer reunion versus novel social investigation.

To investigate OT cell activity in response to familiar and novel peers in a species that forms selective peer relationships, we studied meadow voles (*Microtus pennsylvanicus*) across different social environments, as well as across photoperiods that impact social behavior. Meadow voles are North American rodents that live in social groups and form selective social bonds with peers during winter months^61^. Females of this species are aggressive and territorial during the summer breeding season, but in winter, these territories collapse and voles come together to live in social groups^62,63^. This seasonal ‘switch’ in sociality can be induced in the lab by exposing animals to short, winter-like day lengths (10 h light) versus summer-like long day lengths (14 h light)^61^. Females housed in short days cohabit with other voles more often and in larger groups than long day-housed voles^64^. Short day-housed ‘social’ voles also exhibit selective preferences for familiar same-sex peers within 24 h of cohousing^65,66^, and work harder to access a familiar peer partner than a novel conspecific^25^. Oxytocin infusion can enhance or impair the selectivity of huddling preferences for a peer partner versus a stranger, acting in different brain regions^23,24^.

Behavioral changes with day length coincide with reorganization of the endocrine and nervous system as meadow voles enter a reproductively quiescent state in short day lengths^66–69^. Neural oxytocin receptor density is elevated in short days versus long days across multiple brain regions including the central nucleus of the amygdala and hippocampus^23,69,70^. Whether or not OT production in the PVN is also affected by day length has not been assessed.

To understand how social interactions with familiar and novel individuals differentially impact OT signaling, we assessed OT neuron activity in social (short day length-housed) meadow voles in response to three conditions: separation from a peer partner, reunion, and introduction to an unfamiliar peer (experiment 1). In experiment 2, we measured changes in corticosterone secretion across the same social manipulations used in experiment 1 to determine whether the corticosterone release patterns paralleled OT cell activation. Finally, to understand the potential role of OT in seasonal changes in group living, we assayed OT immunoreactivity in the PVN across photoperiods that correspond with changing social behavior in female meadow voles (experiment 3). Together these results inform our understanding of the impacts of environmental manipulations on OT signaling, and provide a basis for comparisons across species.

## Results

### Experiment 1: Social setting and OT and cFos immunoreactivity

**Fig. 1:**
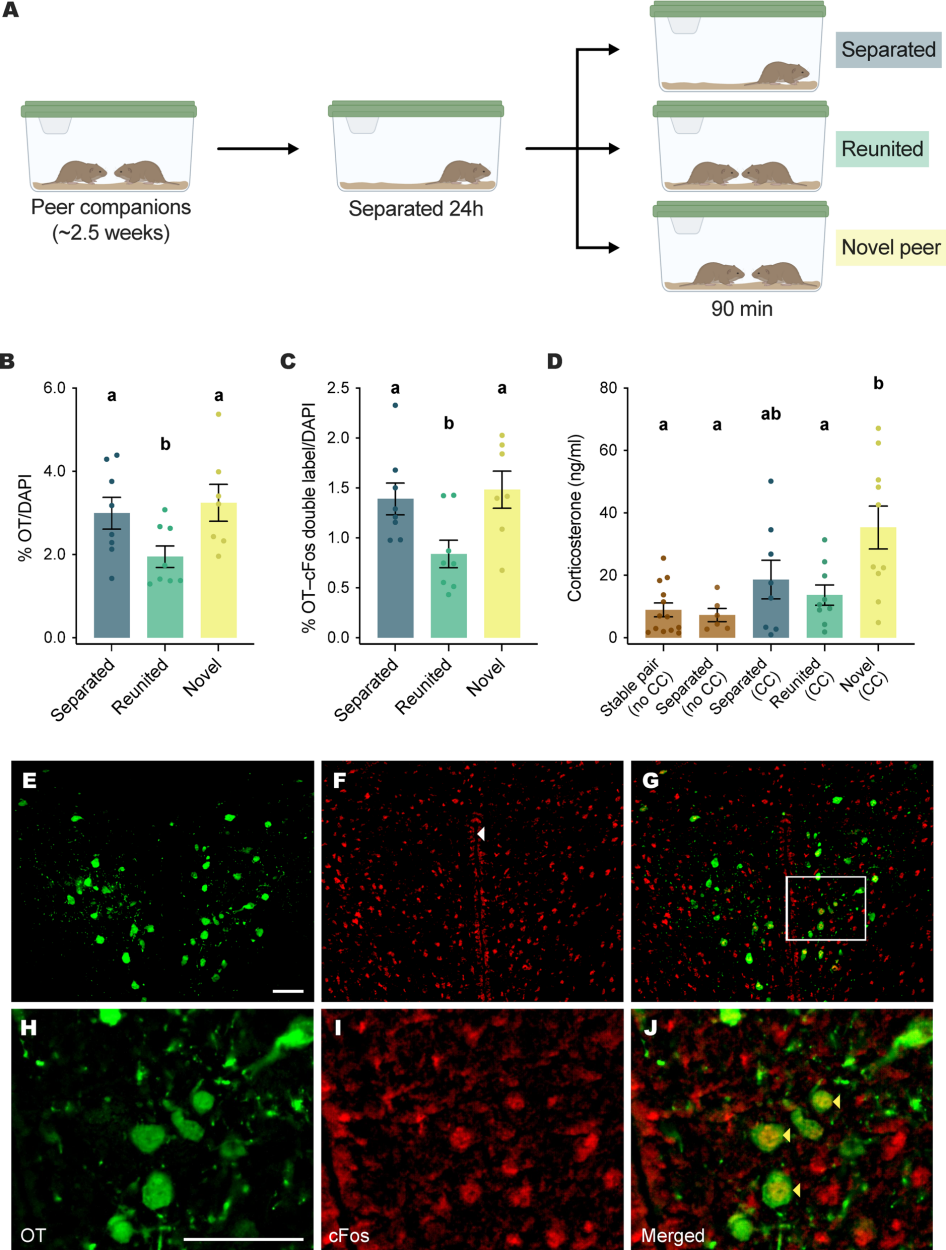
(**A**) Timeline of separation and groups. Voles were paired with an unrelated peer companion in adulthood for 2–2.5 weeks prior to separation for 24 h. All voles were then placed into a clean cage alone (‘separated’), with their original peer companion (‘reunited’), or with an unfamiliar peer (‘novel’). (**B**) OT cell counts in the PVN (relative to total cell count assessed with DAPI imaging) were decreased in voles reunited with their peer partner relative to those that remained separated or those paired with a novel same-sex vole. (**C**) OT/cFos double labeling (relative to total cell count) was reduced in reunited voles relative to other groups. (**D**) Plasma corticosterone in stable, unmanipulated pairs and separated controls (brown), as well as in the three post-separation manipulations used in experiment 1 (blue, green, and yellow bars). CC indicates groups receiving a cage change matching experiment 1. Corticosterone was elevated in the novel condition, but did not differ between baseline and reunited voles. Letters denote significant differences between groups. (**E**) Oxytocin positive cells in the PVN (20× magnification). (**F**) cFos immunolabeling in the same section. The white arrow denotes the position of the third ventricle (**G**). Overlay of A and B showing colocalization of cFos and oxytocin immunoreactive cells. (**H**)–(**J**) Close-ups of boxed region from images in (**E**)–(**G**). Scale bars 50 µM.

OT and cFos immunoreactivity were assessed in short day length-housed (social) voles following a separation and reunion paradigm (Fig. 1A). Female meadow voles from long-term (> 2 week; 14–19 day) peer pairs were separated into individual housing for 24 h. Following isolation they were placed in a clean cage in one of three groups that: (a) remained separated, (b) were reunited with their peer partner, or (c) were introduced to a novel peer (7–8/group; Fig. 1A). 90 min after manipulation, brain tissue was collected for fluorescent immunohistochemistry as described in the methods.

Oxytocin labeling was present in the PVN and SON, and cFos binding was widely dispersed throughout the PVN and surrounding regions. Oxytocin labeling and OT/cFos double labeling were manually scored in the PVN and related to total (DAPI) cell count in each section.

One-way ANOVAs indicated significant differences in both %OT/DAPI cell count (*p* = 0.04, F = 3.6) and %OT/cFos double labeling/DAPI (*p* = 0.0196, F = 4.8) in the central PVN between groups (Fig. 1B,C). The same group differences were also present in unscaled cell counts (Supplementary Fig. S1). OT positive cell count (Fig. 1E,H, Fig S1A) and %OT/DAPI (Fig. 1B) were significantly elevated in the ‘novel’ versus the ‘reunion’ group (*p* = 0.0087, t = 2.9; *p* = 022, t = 2.5 respectively) and in the ‘separated’ versus the ‘reunion’ group (*p* = 0.022, t = 2.4; *p* = 0.05, t = 2.1). OT/cFos double labeling (Fig. 1G,J, Fig. S1) and %double labeling relative to cell count (Fig. 1 C) were also significantly higher in the ‘novel’ group versus the ‘reunion’ group (*p* = 0.0033, t = 3.4; p = 0.011, t = 2.8) and the ‘separated’ versus the ‘reunion’ group (*p* = 0.0069, t = 3.0; *p* = 0.021, t = 2.5).

### Experiment 2: Social setting and peripheral hormone secretion

Because PVN OT neuron labeling was highest in both separated and novel social interaction conditions, both of which may be more stressful than reunion, we assayed corticosterone levels to infer whether OT neuron activity might be part of a stress-coping response. Corticosterone concentration was assessed in a new cohort of animals that included the three experimental conditions used in experiment 1. We also assessed corticosterone in stable, unmanipulated pairs and separated controls that did not receive a cage change to assess whether cage change or separation induced elevations in corticosterone that might predict OT release.

Subjects were co-housed in same-sex pairs for a minimum of two weeks prior to manipulation, with no effect of co-housing duration beyond that interval. One-way ANOVA revealed significant differences in corticosterone levels between groups (Fig. 1D; *p* = 0.0005, F = 6.2). Post-hoc tests (Tukey–Kramer HSD) indicated that corticosterone levels in voles introduced to a novel vole were higher than all other groups except the separated voles who experienced a cage change. Post-hoc pairwise comparisons between the three groups whose methodology matched experiment 1 revealed significantly higher corticosterone levels in the ‘novel’ versus the ‘reunion’ group (*p* = 0.0017, t = 3.4) and significantly higher corticosterone in the ‘novel’ versus ‘separated’ group (*p* = 0.016, t = 2.5).

**Fig. 2:**
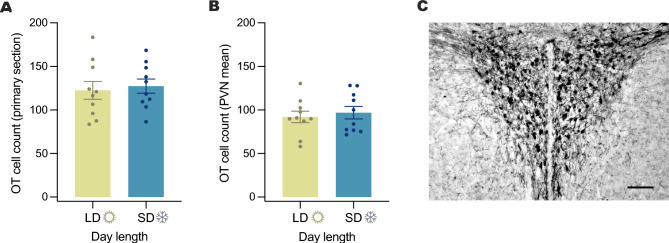
OT immunoreactive cell counts did not differ by day length in (**A**) a specific section of the PVN selected for robust labeling across brains, or (**B**) across sequential averaged sections collected through the PVN. (**C**) representative image of OT immunoreactivity in the key section. Scale bar 100 µM.

We also conducted a pilot assay of serum OT levels in separate animals replicating the three groups used in OT immunohistochemistry to assess whether low OT cell labeling in the PVN was associated with lower peripheral OT release, or was a potential consequence of high peripheral release of OT from these cells. While there were no significant differences in this small sample, voles in the reunited group had the lowest peripheral OT levels (Figure S2), paralleling immunohistochemistry findings (Fig. 1B).

## Experiment 3: Day length and OT immunoreactivity

To determine whether OT production changes concomitant with seasonal changes in sociality, DAB immunohistochemistry was used to quantify OT positive cell counts in long day and short day-housed meadow voles. OT positive cells were observed in the paraventricular and supraoptic nuclei of the hypothalamus, with dense fibers between these regions (Fig. 2C, supplementary Fig. S3B), as well as projecting to other brain regions. There were no significant differences in OT cell count in the PVN by day length either in the representative section (Fig. 2A; *p* = 0.72, t = 0.37) or across the PVN (Fig. 2B; *p* = 0.62, t = 0.50).

## Discussion

Familiar and novel social companions represent very different social stimuli, especially for meadow voles, who demonstrate selective partner preferences for familiar group members in short day lengths^23–25,66,71,72^. This difference was evident in %OT cell counts and %OT/cFos colocalizations in the PVN relative to cell count following 24 h separation: pairing with a novel social stimulus resulted in higher immunoreactivity than did reunion with the peer partner.

Intriguingly, voles who remained separated also had higher %OT and %OT/cFos colocalization than did voles reunited with their partner. Prior work in mice and prairie voles has shown elevated OT cell counts and OT/cFos colabeling in the PVN after 1–4 weeks of separation^42,44^. Although our separation was much shorter, it’s possible our higher OT in the separation group represents upregulation of PVN OT/cFos positive neurons during the separation condition. Future studies could include a no-separation control group to confirm this finding. These findings are consistent with the idea that OT cell activity is high during incubation of craving and is ameliorated during reunion but not during a novel social encounter in meadow voles.

Although we expected to see differences in OT/cFos colabeling after reunion versus novel pairing, impacts on the OT cell count 1.5 h after social manipulation were unexpected. This timing suggests that differences between the novel and reunion groups do not result from novel OT synthesis, but perhaps more dynamic release or regulation. For example, decreased OT immunofluorescence might occur following substantial OT release/depletion from these neurons. Exposure to CO2 euthanasia can substantially increase OT release into the bloodstream^73^, and while this was matched across groups, it’s possible that groups were differentially affected based on the readily releasable pool of neuropeptide. Alternatively, OT that is being mobilized for release may become more available to immunofluorescent detection. To assess how OT immunofluorescence corresponds to peripheral release we added a small-scale study measuring serum OT following the social manipulations used in experiment 1. These findings did not indicate greater OT release in the reunion group, and, while not significant, hint at both greater OT release to the periphery in the novel condition and greater detection of OT neurons in this group. However, OT was only assessed at 90 min post-manipulation, matching earlier brain collection. While we have seen evidence of lasting (2–3 h) elevations to OT following manipulations in our other studies (unpublished data), OT could have been released and degraded prior to our sampling timepoint. To further explore the dynamics of peripheral OT release in response to social interaction, we will need to assess OT at different time intervals following social manipulations.

### Plasma corticosterone following social manipulations

One potential explanation for elevated OT cell immunoreactivity in both the separated and novel groups is that OT neurons are active during times of stress. A recent study found that OT neurons are much more highly activated by both social and non-social stressors than by social interaction^35^. We therefore asked whether corticosterone release patterns matched OT/cFos cell labeling. Corticosterone was significantly elevated in the novel versus familiar condition, paralleling OT immunoreactivity and consistent with the hypothesis that OT neuron activity parallels stress exposure.

While novel pairing significantly increased corticosterone concentrations, such pairings after brief isolation eventually lead to new peer social bonds after 24 h^24,71,72^. Meadow voles are subject to high predation; to maintain winter groups, voles integrate new group members throughout the winter^74,75^ and form new relationships with multiple peers in short day lengths in the lab^23,64,71,72^. While meadow voles are not affiliative toward strangers, they rarely show aggression in social interaction tests with novel peers, unlike prairie voles^76^. Our novel pairing + cage-change condition, and the resulting increase in corticosterone, are thus equivalent to conditions in which social bonds go on to develop. However, forced swim, and its concomitant more extreme corticosterone increase (to ~ 5 times baseline concentration), impairs social bonding in meadow voles^72^. This indicates that there are levels of corticosterone upon social introduction that are tolerated to facilitate new bond formation.

It is possible that corticosterone release coupled with OT neuron activation during separation and novel pairing are priming animals for new bond formation. OT signaling significantly accelerates bond formation with mates or peers in prairie voles^77,78^, and can enhance peer relationship formation in meadow voles^23^. Future studies will ideally monitor real-time OT release dynamics in the brain during social interaction with bonded peer partners and novel individuals. The development of sensors such as GRAB-OT^79^ that enable imaging OT release in vivo during social behaviors promises to make major contributions to our understanding of situation-dependent activity of OT neurons.

Corticosterone was lowest in unmanipulated pairs, and in separated animals who did not undergo a cage change shortly before euthanasia. Measuring corticosterone 24 h after a separation did not show elevations compared to paired voles. While these groups were not significantly different from 24 h separation with a cage change, cage changes significantly increase heart rate in rats^80^, and corticosterone levels in mice^81^, and we saw a trend toward the same phenomenon in voles. Within groups parallel to experiment 1 (in which all subjects were placed in a clean cage), voles who underwent a cage change but were reunited with a partner showed intermediate corticosterone levels between voles who underwent a cage change alone and those paired with a novel vole. Reunion with a familiar animal may buffer the mildly stressful effects of cage change.

### Photoperiodic influences on oxytocin signaling

Major reorganization of OTR by day length has been previously reported in meadow voles across multiple studies^23,69,70^, with higher OTR density in the brains of short day meadow voles across several regions, including the central amygdala and hippocampus. It was unknown, however, whether OT production changes with photoperiod in this species as it does in female California mice^82^. We found no differences in OT positive neurons across day lengths. When considered in concert with prior data on OTR densities, this finding indicates that seasonal variation in OT signaling is mediated through receptor densities. This is consistent with hypotheses that neuropeptide receptor densities, which vary markedly across species and life history states^83–85^ are likely candidates for evolutionary tuning knobs^86,87^. In essence, the density and distribution of neuropeptide receptor expression is evolutionarily labile and capable of altering circuit function, while OT production and projection across the brain are less easily modified and more highly conserved. While differences in OT production were not evident across day lengths, moment to moment variation in OT production and release patterns with experience, as found in experiment 1, may well differ between day lengths. This could be assessed using optical sensors in slice preparation. For example, brain tissue from short day- and long day-housed meadow voles differs in dopamine release in response to electrical stimulation^88^, and similar assessments across day length could be performed with a selective OT sensor that works in voles^89^.

## Conclusions

Decades of research on oxytocin have revealed its critical importance for wide-ranging social processes in mammals. In studying meadow voles that form selective relationships with known peers, we find significant differences in OT immunoreactivity between interactions with novel individuals and familiar companions. These differences are consistent with OT neuron activity during social and non-social stress. Seasonal changes in OT signaling appear to be mediated through receptor density rather than OT production, although activity-dependent differences in OT release are also likely.

## Materials and methods

### Animal subjects

Subjects were female meadow voles (*Microtus pennsylvanicus*), as only females exhibit strong seasonal variation in peer social behavior^71,90^. Meadow voles were bred locally in our colony, which was periodically outbred with meadow voles collected from Hampshire County, MA (Massachusetts Division of Fisheries and Wildlife permit 012.14SCM). On postnatal day 18–20, experimental voles were weaned into short day (SD) or long day (LD) lengths consisting of either a 10 h:14 h light:dark or 14 h:10 h light:dark cycle, respectively. Temperature was maintained at 68 +/− 3 °F. Voles were housed in clear plastic cages (17.5″ × 8.5″ × 6″), containing aspen bedding, Envirodri nesting material (Shepherd Specialty Papers), and an opaque plastic tube. All animals received Mouse Chow and water ad libitum*,* in addition to weekly fruit and vegetable supplements. All procedures were governed by the National Institute of Health *Guide for the Care and Use of Laboratory Animals*, and were approved by the Institutional Animal Care and Use Committees at Smith College and The University of California, Berkeley. All methods were performed in accordance with the relevant guidelines and regulations, and experimental design and analysis conformed to ARRIVE guidelines.

### Experimental design and timelines

#### Experiment 1: Social setting and OT immunoreactivity

Female voles were weaned into single housing in short day lengths, and later paired with a non-sibling female peer partner at 69 (± 17) days of age for 16 (± 3) days. Between 11:00am and 12:00 pm, animals were separated from their partners into solitary housing in a clean cage with food and water for 24 h. After separation, animals remained alone (separated; n = 8), were reunited with their familiar peer partner (reunion; n = 8), or were paired with a novel, non-sibling partner (novel; n = 7). All treatments involved placement into a clean cage, and animals were transferred via PVC tube. 1.5 h after placement in their experimental condition, voles were euthanized with CO_2_ and brains were removed for fluorescent immunohistochemistry.

#### Experiment 2a: Social setting and corticosterone

In a new set of subjects, corticosterone levels were examined across the social settings described in experiment 1 as well as two additional groups. Females were cohoused with a non-sibling peer for a minimum of two weeks. Trunk blood was collected at approximately 1 pm in all subjects, following euthanasia by rapid decapitation (see blood collection for details) at 91 (+/− 8) days of age. Subjects in the separated (n = 8), reunion (n = 9), and novel (n = 10) conditions all underwent 24 h separation followed by placement into a clean cage alone or with their respective partner depending on group. Samples were collected 90 min after this manipulation. A no-handling control group (baseline-no cage change, n = 13), as well as a separation only group (n = 6, separation-no cage change) were included to distinguish any effects of separation or cage change on corticosterone levels.

#### Experiment 2b: Social setting and serum oxytocin

In additional subjects, serum OT levels were measured between 10 and 11am across the social manipulations described in experiment 1. 90 min following social manipulation, animals were anesthetized with isoflurane for ~ 2 min and trunk blood was collected (see blood collection) and serum was stored at − 80 °C until the time of OT enzyme immunoassay.

#### Experiment 3: DAB OT immunohistochemistry across day lengths

A third set of meadow voles (n = 10 short day, 10 long day) was euthanized under isoflurane at 61 (+ /- 16) days. Brains were extracted and immersion fixed, then sliced and placed in cryoprotectant at − 20 °C as in experiment 1.

### Fluorescent immunohistochemistry

Brains were immersed in 5% acrolein (Alfa Aesar, Ward Hill, MA) in 0.1 M PBS for four hours, then changed into fresh acrolein solution overnight. Brains were rinsed in phosphate buffer (PB), and immersed in 0.1 M PB/30% sucrose solution until they sank (2–3 days). Brains were sliced on a microtome at 40um into four parallel series (160um between adjacent sections). Free-floating sections were stored in cryoprotectant (30% sucrose, 1% polyvinylpyrrolidone in phosphate buffer and ethylene glycol) at − 20 °C until processing. Free-floating sections containing the PVN were washed 6 × 5 min in PBS, followed by a 15-min immersion in 0.1% sodium borohydride in PBS. Sections were washed 3 × 5 min in PBS, then blocked in a PBS solution containing 0.3% Triton-X (PBS-T) and 5% NGS for 30 min. Sections were incubated in a PBS-T solution containing monoclonal mouse anti-OT primary antibody (MAB5296, lot 2136574, EMD Millipore Corporation, 1:10,000 dilution) and polyclonal rabbit anti-cFos primary antibody (sc-52, lot D0214, Santa Cruz Biotechnology, 1:100) for 1 h at RT, followed by 48 h on a plate shaker at 60 rpm at 4°C. Following the primary antibody incubations, sections were washed 3 × 5 min in PBS, and incubated in secondary antibody for 3 h on a plate shaker (60 rpm at 4 °C). The incubation solution consisted of PBST with 1% NGS and 1:1000 dilutions of the secondary antibodies Alexa Fluor 488 goat anti-mouse antibody (A11029, lot 1306597, Life Technologies) and Alexa Fluor 568 goat anti-rabbit antibody (A11036, lot 1301874, Life Technologies). Sections were then washed 2 × 5 min in PBS-T, followed by a 5 min PBS wash. Sections were mounted onto gelatin-subbed slides from 0.75X PBS, dried, and cover-slipped using Vectashield H1500 Hard-set Mounting Medium with DAPI (Vector Laboratories). Slides were placed in dark storage and imaged within 48 h. Additional sections were used for negative controls (no primary antibody, no secondary antibody, or neither).

Sections were photographed at 20× magnification under the wide ultraviolet, wide blue, and wide green filter cubes for DAPI, Alexa 488 and Alexa 568 fluorophores respectively. Exposure times were 16.667 ms for DAPI, 111.11 ms for Alexa 488 (OT secondary) and 50 ms for Alexa 568 (cFos secondary). Starting with the anterior commissure fusion, slices from each brain were ordered and aligned to an atlas. Four slices from each brain were quantified—centered on anatomical markers corresponding to plate 26 of the Rat Atlas in Stereotaxic Coordinates^91^ and set 22 (OC + 1000 uM) of the Prairie Vole Brain Atlas^92^. Cell counts were manually scored without knowledge of treatment group following background subtraction using ImageJ/Fuji, and scaled relative to counts of cells stained with DAPI. The central section was used for statistical comparisons as it had reliable labeling and was present in all brains. Averaged counts across the 4-section span of the PVN were also assessed and yielded similar findings (see figure S1C,D and supplementary data file).

### Blood collection

Blood for corticosterone assays was collected into EDTA treated microvials within 2 min of initial cage disturbance. Blood for oxytocin EIAs was collected into untreated tubes. Blood samples were centrifuged at 4 °C at 3500×G for 20 min, and plasma or serum were collected and stored at − 80 °C until quantification.

### Corticosterone quantification

Corticosterone ELISA assay was conducted at a 1:20 dilution following kit protocols (ADI-900–097, lot 12041401D, Enzo Life Sciences), and plates were read on a SpectraMax M5 plate reader at 405 nm. Samples were not reported if they fell outside the detection range of the standard curve and were excluded if they met our pre-determined criteria of > 30% coefficient of variability between replicates (n = 1) or ± 3SD from the global mean (n = 1). Sample sizes listed in experiment 2 are those remaining after exclusion. Mean intra-assay %CV (between replicates) was 8.9%, and inter-assay %CV (between plates) was 8.7%.

### OT quantification

Oxytocin ELISA (500440; Cayman Chemical Batch 0807811) was conducted at ¼ dilution (1 part sample: 3 parts assay buffer) following kit protocols. The plate was read at 405 nm with an Absorbance 96 plate reader (Byonoy GmbH). All samples met our pre-determined criteria of > 30% coefficient of variability between replicates and were included. Mean %CV between sample replicates was 5.80%.

### DAB immunohistochemistry

Sections were washed 3 × 5 min in TBS, followed by a 30-min incubation in 0.05 M sodium citrate. Sections were again washed 3 × 5 min in TBS and then incubated in 0.1 M glycine for 30 min. Sections were washed 3 × 5 min in TBS and incubated in a blocking solution of 0.4% TBS-Triton-X, 10% normal goat serum (NGS) and 1% hydrogen peroxide. Sections were incubated overnight at room temperature with the primary antibody, guinea pig anti-oxytocin (T-5021, lot A09110, Peninsula Laboratories, LLC) in TTG (TBS, 10% Triton-X, NGS) at a final dilution of 1:15,000. Sections were washed 6 × 5 min in TTG and incubated at room temperature in the biotinylated goat anti-guinea pig (BA-9200, lot W0726, Vector Laboratories) secondary antibody for 90 min at a 1:500 dilution. Sections were washed 3 × 5 min in 1× TBS with 0.4% Triton-X (TBS-T), and incubated in ABC solution (PK6100, Vectastain ABC-Elite Standard Kit, Vector Laboratories) for 90 min (1:100). Sections were rinsed 3 × 5 min in 1X TBS and stained in DAB HRP substrate for one minute (SK-4100, DAB Peroxidase (HRP) Substrate Kit, 3,3′-diaminobenzidine, Vector Laboratories). Before mounting, sections were rinsed 3 × 5 min in TBS. Sections were mounted on dry slides using Permount. Images were acquired at 10× magnification for scoring and 4× magnification for anatomical reference. Images were scored in ImageJ (nih.gov) using the Cell Counter plugin (Kurt De Vos, University of Sheffield) by two different researchers blind to experimental conditions. Four sections containing the PVN were quantified from each brain, as described for fluorescent immunohistochemistry.

### Statistical analyses

Statistical analyses were performed in JMP (SAS Institute Inc) and Prism (Graphpad), and a threshold of *p* < 0.05 was considered statistically significant. Comparisons between groups were made by one-way ANOVA followed by Tukey’s HSD. Day length differences in OT production were examined using *t*-tests between groups.

## Supplementary Information

Supplementary Information 1.

Supplementary Information 2.

## Data Availability

All numeric data are included in this published article and its supplementary information files. Raw data (e.g. image sources) are available from the corresponding author on reasonable request.
