# Supplementary material for: Reunion with a peer partner reduces PVN oxytocin neuron immunoreactivity in socially selective voles
Source: Sci Rep. 2025 Sep 29;15:33523. doi: 10.1038/s41598-025-17920-3 (PMC12480597; doi:10.1038/s41598-025-17920-3)
Supplement: Supplementary file 2 — Supplementary Information 2. [file 41598_2025_17920_MOESM2_ESM.pdf]

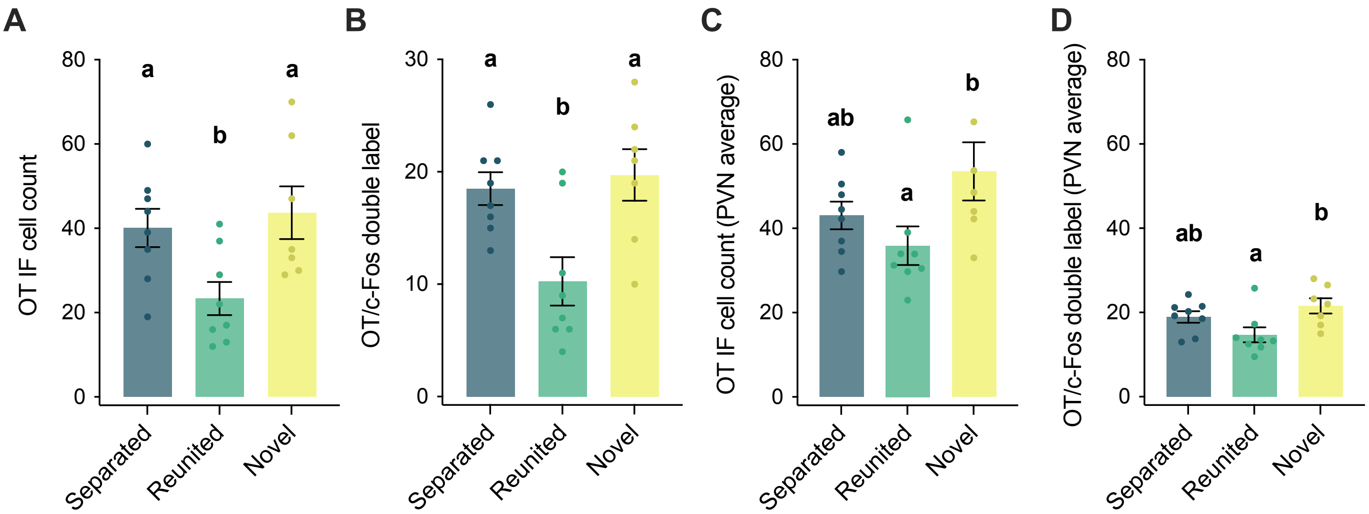

Figure S1. Counts of OT immunofluorescent cells (**A**) and OT and cFos double labeled cells (**B**) in the anterior PVN. **C** and **D**: corresponding cell counts averaged across four PVN sections spaced 200μM apart. Letters denote significant differences between groups.

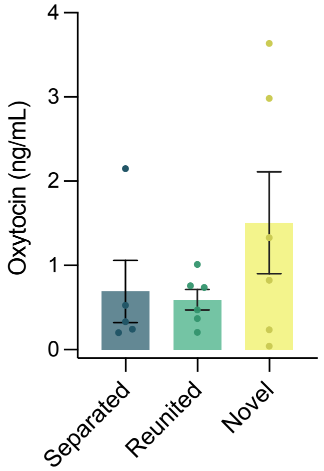

Figure S2. Pilot assay of serum OT following the social manipulations performed in experiment 1 (with different animals). Voles in the reunited group did not show higher peripheral OT levels, indicating lower PVN OT neuropn labeling in this group was unlikely to be the result of extensive release into circulation.

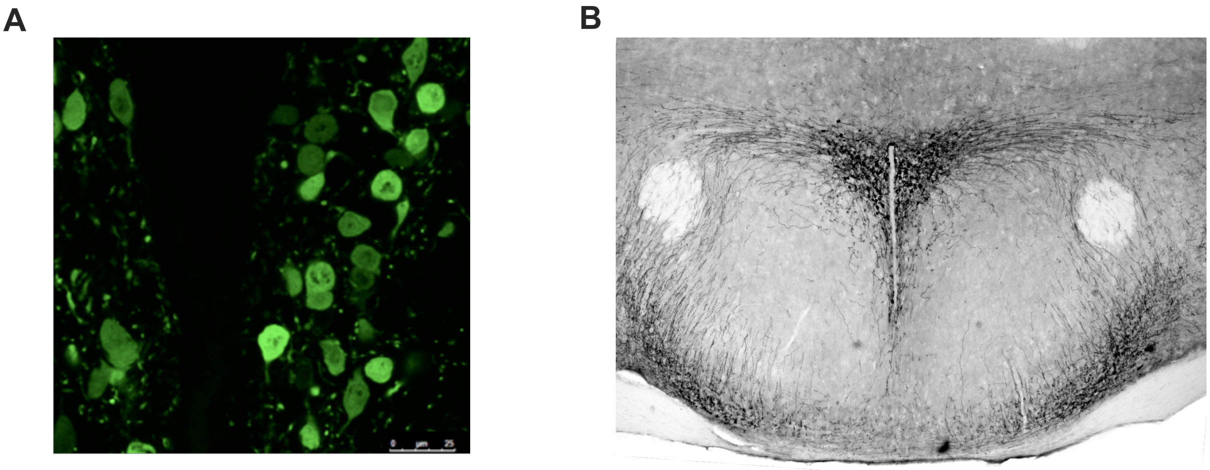

Figure S3. A. Confocal image (63x) of OT immuofluorescent cells with original scale bar (25μM, image subset shown). B. Lower resolution (4x) image of DAB OT labeling with PVN, SON, and OT fiber pathways.
